# Supplementary material for: Identifying the Compounds of the Metabolic Elicitors of Pseudomonas fluorescens N 21.4 Responsible for Their Ability to Induce Plant Resistance
Source: Plants (Basel). 2020 Aug 12;9(8):1020. doi: 10.3390/plants9081020 (PMC7463883; doi:10.3390/plants9081020)
Supplement: Supplementary file 1 [file plants-09-01020-s001.pdf]

Supplementary files

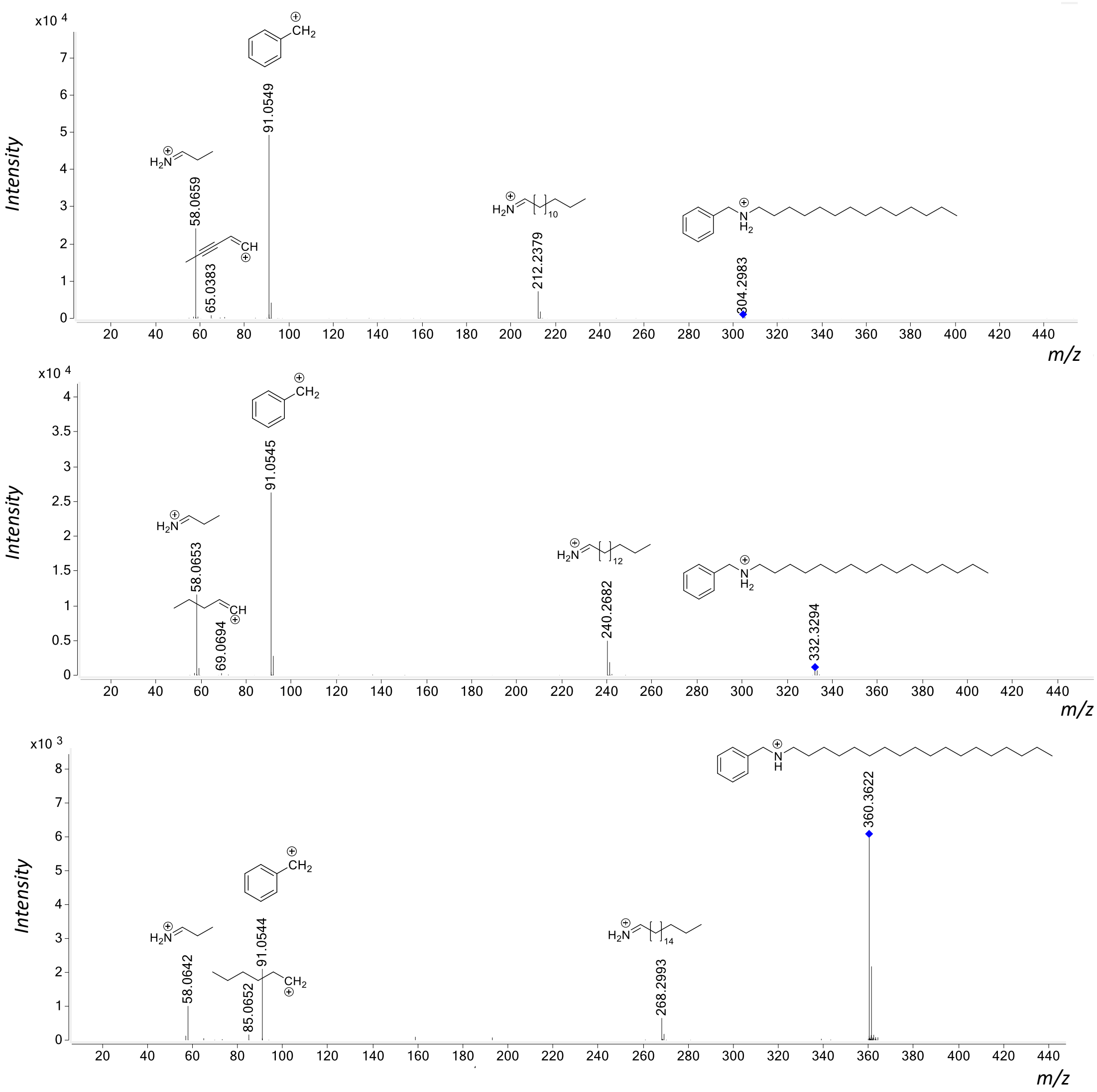

Figure 1: Experimental MS/MS spectra for the aryl alkylamines detected in the extract at the collision energy (30 eV) in positive.

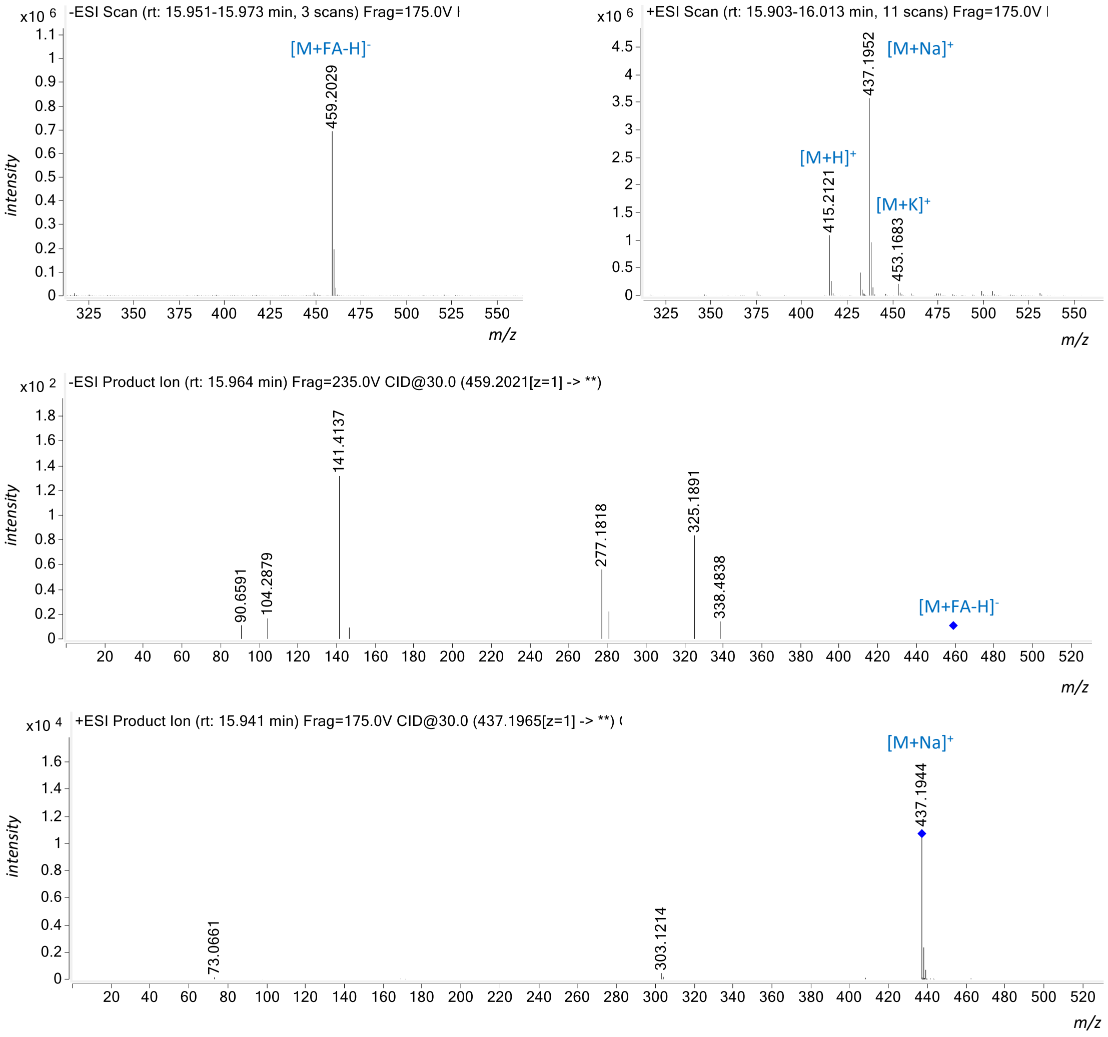

Figure 2: Experimental MS/MS spectra at the collision energy (30 eV) in positive and negative.
